# Mycosis fungoides with large cell transformation associated with oral deucravacitinib

**DOI:** 10.1016/j.jdcr.2024.09.003

**Published:** 2024-09-24

**Authors:** Devyn Zaminski, Dolly Taiwo, Shane A. Meehan, Jason Weed

**Affiliations:** a The Ronald O. Perelman Department of Dermatology, NYU Grossman School of Medicine, New York, New York; b Department of Dermatology, Mount Sinai Health System, New York, New York

**Keywords:** cutaneous t-cell lymphoma, deucravacitinib, mycosis fungoides, psoriasis vulgaris

## Introduction

Mycosis fungoides (MF) is the most common subtype of cutaneous T cell lymphoma and is characterized by a proliferation of malignant T cells in the skin with epidermotropism and wide clinical and histologic variation. In the early stages, it can present with erythematous patches or plaques that share features with other papulosquamous dermatoses.^1^ Estimates of 20% to 55% of advanced MF cases show large cell transformation, a histological feature associated with clinical development of nodules or tumors, and a 5-year survival rate of less than 20%.^2^ MF is characterized by immune dysregulation and has been noted to occur in patients on immunosuppressive therapies, including tumor necrosis factor-alpha inhibitors and other agents.^3^ Janus kinase (JAK) inhibitors are immunosuppressive agents increasingly used in dermatology; among these, the tyrosine kinase 2 (TYK2) inhibitor deucravacitinib is approved for use in moderate-to-severe plaque psoriasis.

In this case report, we present a 61-year-old woman who had been clinically diagnosed with plaque psoriasis and initiated on deucravacitinib. Three months after starting deucravacitinib, she developed tumors within these plaques. Histology revealed MF with large cell transformation. To our knowledge, this report describes the first published case of a diagnosis of mycosis fungoides with large cell transformation following deucravacitinib use.

## Case report

**Fig 1:**
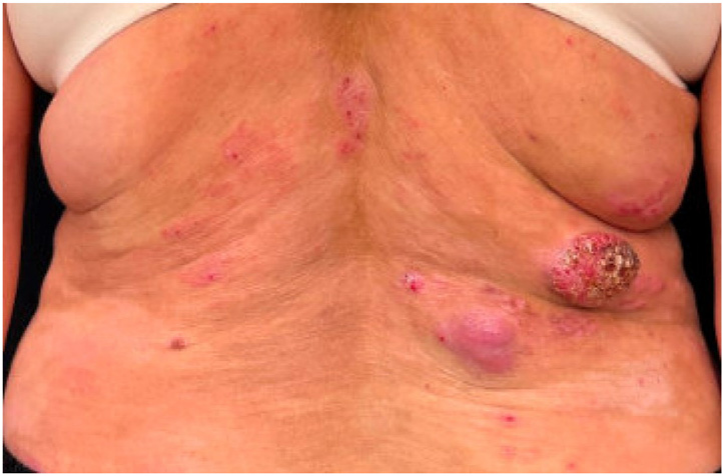
Mycosis fungoides with large cell transformation in a superficially crusted and centrally eroded 4 cm tumor on the right lower back, appearing after the patient underwent 3 months of deucravacitinib therapy. Secondary pink tumor located inferiorly and medially, with nearby scaly patches and thin plaques.

**Fig 2:**
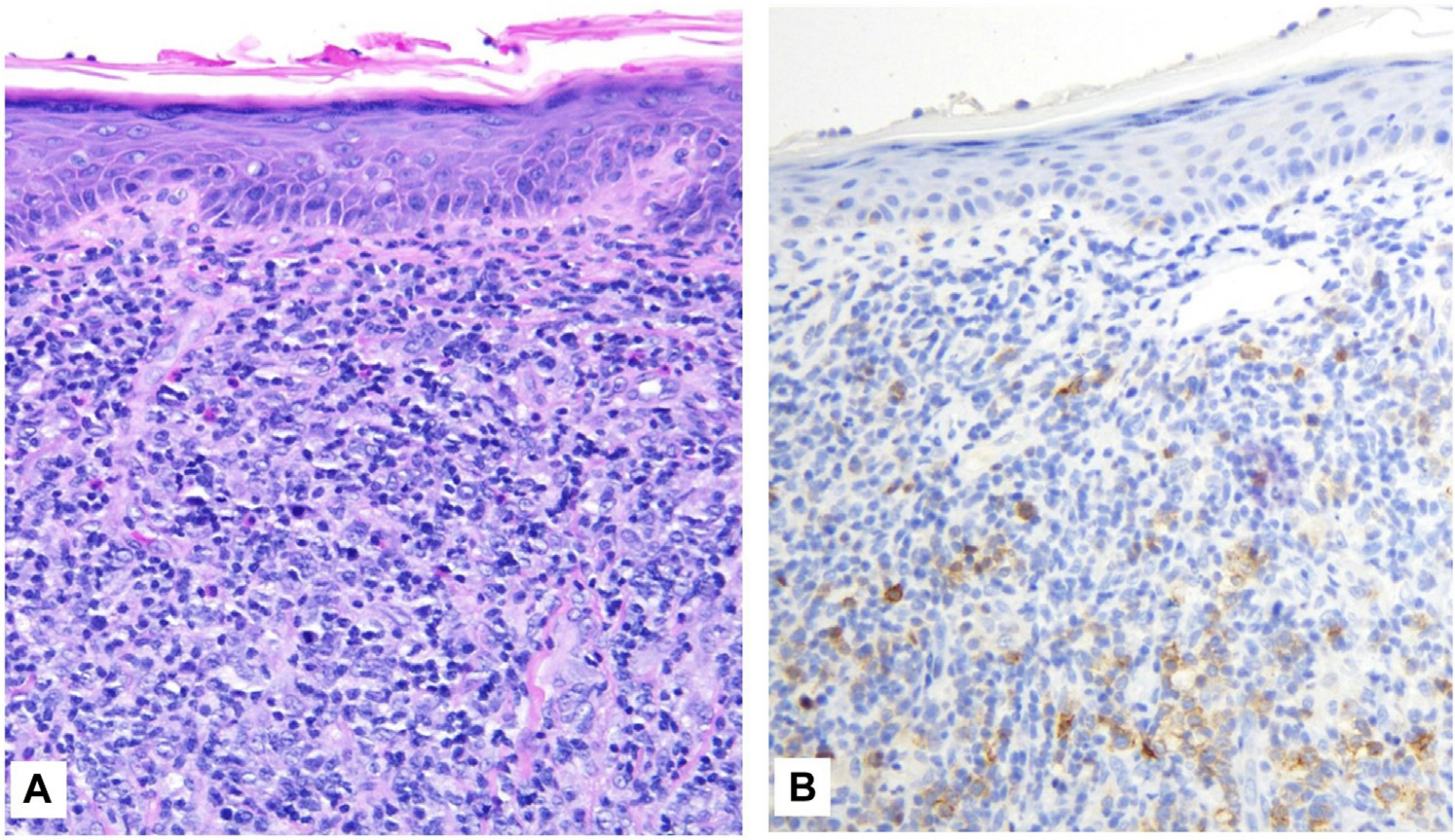
Skin biopsy of mycosis fungoides with large cell transformation revealing (**A**) dense infiltrate of large lymphocytes, many of which have irregular nuclear contours. **B,** Many of the larger lymphocytes react for CD30.

A 61-year-old woman presented to an outpatient dermatology clinic for evaluation of pruritic plaques localized to the lower back for 4 months (estimated body surface area <10%) and was clinically diagnosed with plaque psoriasis. Her medical history was notable for hypertension and hyperlipidemia managed with atorvastatin and losartan. She was prescribed topical calcipotriene 0.05% cream, topical taparinof 1% cream, and oral deucravacitinib, but within 3 months round tumors arose among these plaques. A 4 mm punch biopsy of a tumor on the right lower back showed findings consistent with plaque stage mycosis fungoides. Deucravacitinib was stopped, and she was referred to our institution. Clinical exam revealed a 4 cm erythematous superficially scale-crusted tumor on the right lower back, with an adjacent 3 cm tumor, as well as scaly plaques and erythematous patches on the back, trunk, and extremities (Fig 1). Complete blood counts, electrolyte panel, lactate dehydrogenase, and flow cytometry on peripheral blood were normal. Topical taparinof and calcipotriene were stopped. A repeat punch biopsy of the larger tumor demonstrated a dense dermal infiltrate of medium-sized and large lymphocytes, many with irregular nuclear contours, positive staining for CD3 and CD30, partial loss of CD7 expression, elevated Ki-67 expression, and without noted folliculotropism or follicular mucin (Fig 2). A positron-emission tomography scan revealed hypermetabolic regions in the lower back with supra- and infra-diaphragmatic lymphadenopathy. The patient underwent radical surgical tumor resection of the 2 adjacent tumors on the lower back for immediate debulking and started oral low-dose methotrexate and topical clobetasol. She developed one nodule on the left arm, which was resected, and she then transitioned to oral bexarotene. Over the subsequent months, she developed new cutaneous tumors and transitioned to brentuximab.

## Discussion

Herein, we report the case of a patient clinically diagnosed with psoriasis and treated with deucravacitinib who developed tumors within her plaques and was found to have MF with large cell transformation. MF may share clinical features with common skin eruptions, including papulosquamous dermatoses such as psoriasis, which affects 1% to 3% of the world’s population and, as such, is one of the most common chronic dermatoses.^4^ In contrast to psoriasis, MF has a lower incidence of 5.42 per million people^5^ and only a small subset of patients (often with clinically advanced MF) undergo large cell transformation, resulting in a more aggressive clinical course and a 20% 5-year survival rate.^2^

This patient may have presented with psoriasis and later developed MF. Numerous cases have been reported of patients with psoriasis who develop MF after starting immunosuppressive therapies, such as anti-tumor necrosis factor agents.^3^ A review of clinical trials of deucravacitinib found that patients developed malignancies (including lymphoma) at a rate of 0.3 per 100 person years.^6^ Alternatively, the patient’s initial papulosquamous eruption may have been psoriasiform mycosis fungoides, as the patient had neither a family history of psoriasis nor lesions in a distribution strongly suggestive of psoriasis vulgaris, inverse psoriasis, or other well-characterized psoriasis variants and was not biopsied prior to treatment. Clinically, psoriasiform MF closely mimics psoriasis. Both can present with erythematous, scaly, pruritic skin patches or plaques. In this setting, deucravacitinib could have contributed to the rapid evolution from early plaque MF to advanced MF with large cell transformation. While JAK inhibition has been investigated with promise in a clinical trial^7^ as a therapy for T cell lymphomas, the connection between JAK inhibition and lymphoma pathogenesis is necessarily nuanced; inhibition of the JAK and related proteins may suppress activated pathways in malignant T cells but may also suppress immune pathways critical for the anti-tumor response.^8^ This concern is not limited to TYK2 inhibition. A case of rapid worsening of a psoriasiform dermatitis later revealed to be mycosis fungoides has also been reported with use of ruxolitinib, a JAK1/2 inhibitor.^9^

In conclusion, this case describes a patient who underwent deucravacitinib therapy for 3 months, during which initially pruritic plaques developed into tumors diagnosed as MF with large cell transformation. Physicians should strongly consider obtaining a skin biopsy and using clinicopathologic correlation to diagnose psoriasis before initiating deucravacitinib therapy, as psoriasis and psoriasiform MF can have overlapping clinical presentations.

## Conflicts of interest

None disclosed.

## References

[1] Cerroni L. (2018). Mycosis fungoides—clinical and histopathologic features, differential diagnosis, and treatment. Sem Cutan Med Surg.

[2] Pulitzer M., Myskowski P.L., Horwitz S.M. (2014). Mycosis fungoides with large cell transformation: clinicopathological features and prognostic factors. Pathology.

[3] Dequidt L., Franck N., Sanchez-Pena P. (2019). Cutaneous lymphomas appearing during treatment with biologics: 44 cases from the French Study Group on cutaneous lymphomas and French Pharmacovigilance Database. Br J Dermatol.

[4] Korman N.J. (2020). Management of psoriasis as a systemic disease: what is the evidence?. Br J Dermatol.

[5] Cai Z.R., Chen M.L., Weinstock M.A., Kim Y.H., Novoa R.A., Linos E. (2022). Incidence trends of primary cutaneous T-cell lymphoma in the US from 2000 to 2018: a SEER population data analysis. JAMA Oncol.

[6] Hoy S.M. (2022). Deucravacitinib: first approval. Drugs.

[7] Moskowitz A.J., Ghione P., Jacobsen E. (2021). A phase 2 biomarker-driven study of ruxolitinib demonstrates effectiveness of JAK/STAT targeting in T-cell lymphomas. Blood.

[8] Russell M.D., Stovin C., Alveyn E. (2023). JAK inhibitors and the risk of malignancy: a meta-analysis across disease indications. Ann Rheum Dis.

[9] Blanchard G., Bisig B., de Leval L., Hohl D., Guenova E. (2024). Cytokine-pathway blockers worsen mycosis fungoides masquerading as psoriasis. JAAD Case Rep.

